# Antibody Response after SARS-CoV-2 Infection with the Delta and Omicron Variant

**DOI:** 10.3390/vaccines10101728

**Published:** 2022-10-16

**Authors:** Agata Błaszczuk, Aleksander Michalski, Dominika Sikora, Maria Malm, Bartłomiej Drop, Małgorzata Polz-Dacewicz

**Affiliations:** 1 Department of Virology with SARS Laboratory, Medical University of Lublin, 20-093 Lublin, Poland; 2 1st Clinical Military Hospital with Outpatient Clinic in Lublin, 20-049 Lublin, Poland; 3 Department of Computer Science and Medical Statistics with the e-Health Laboratory, 20-090 Lublin, Poland

**Keywords:** SARS-CoV-2, COVID-19, SARS CoV-2 antibody, NCP, RBD, S2, delta variant, omicron variant, vaccination

## Abstract

The SARS-CoV-2 virus caused a worldwide COVID-19 pandemic. So far, 6,120,834 confirmed cases of COVID-19 with 116,773 deaths have been reported in Poland. According to WHO, a total of 54,662,485 vaccine doses have been administered. New variants emerge that become dominant. The aim of this study was a comparison of antibody level after infection caused by Delta and Omicron variants. The study included 203 persons who underwent mild COVID-19 despite two doses of vaccine. The obtained results indicate that a significantly lower titer was observed in patients with the Omicron variant infection. Therefore, these patients may be at risk of reinfection with new strains of the Omicron variant. Due to the possibility of reinfection, booster vaccinations are necessary. Further epidemiological and clinical studies are necessary to develop new prevention strategies.

## 1. Introduction

SARS-CoV-2 infection and the COVID-19 epidemic are major public health problems worldwide. In Poland, from 3 January 2020 to 16 August 2022, there have been 6,120,834 confirmed cases of COVID-19 with 116,773 deaths reported to WHO. As of 10 July 2022, a total of 54,662,485 vaccine doses have been administered [1].

SARS-CoV-2, a member of the *Coronaviridae* family and of Betacoronavirus genera, has an RNA genome consisting of several non-structural proteins interspaced with structural proteins genes for spike (S), envelope (E), membrane (M), and nucleocapsid (N) [2]. The spike (S) protein, the most important for cell invasion, consists of approximately 1280 nt and contains a 13 nt-long signal sequence and S1 and S2 subunits. S1 is further divided into the receptor binding domain (RBD) and N-terminal domain (NTD). Part of the RBD is a receptor binding motif (RBM) [3,4].

S protein has a key role in viral entry to the cell and makes a potential target for antibodies. The error-prone replication mutation rate in the S gene of SARS-CoV-2 is high [4,5], and mutations in the S gene may influence the affinity of binding to the host cell, its infectivity, and transmissibility [2,3,4,5].

Many new variants appeared and WHO divided those variants based on the level of their current threat to the worldwide community [6]. Alpha, Beta, Gamma, Delta, and Omicron are the current variants of concern (VOC), whereas Lambda and Mu are variants of interest (VOI), and three strains (B.1.1.318, C.1.2., B.1.640) are variants under monitoring (VUM). All those variants, as well as other previously monitored ones, accumulate as mutations, mainly in the S protein gene, which enable the virus to spread faster and more effectively.

In early 2021, the Delta variant, or B.1.617., was first discovered in India and soon was detected all over the world, displacing previous strains [7]. Important features of the Delta VOC are higher transmission rate and viral load in infected cells, shorter incubation period, and noticeably more severe and longer course of disease that significantly overstretched public health care [3,5,6,8,9,10].

The first case of another new variant was detected in November 2021 in southern Africa and Botswana and quickly spread to western Europe [8,11]. It was qualified as VOC [8].

The Omicron variant has a mutation rate 3–4 times higher than previous strains [7,8,9,10,11,12]. “Omicron-positive” patients had milder symptoms, usually including general fatigue, sore throat, mild cough, and slightly raised temperature. New variants may reduce the effectiveness of the vaccines [7,8,9,11,12,13].

In Poland, the wave caused by Delta variant lasted from February 2021 to December 2021, and was then replaced by the new wave of the Omicron variant.

The aim of our study was to check the titers of nucleocapsid protein (NCP), RBD, and S2 antibodies in the studied patients who underwent mild COVID-19 despite two doses of the vaccine. We made a comparison in which patients, depending on the infection with a specific Delta or Omicron variant, had higher or lower antibody levels. Antibody levels were also analyzed by age and sex.

## 2. Materials and Methods

### 2.1. Study Design

Patients tested for SARS-CoV-2 in our laboratory were qualified for the analysis. We sent an invitation to PCR positive patients to participate in the antibody level test. People who agreed to participate in the study completed the questionnaire containing questions on the following topics: SARS-CoV-2 vaccination (dates of vaccine receipt and type of vaccine), hospitalization, or not. Archived nasopharyngeal swab samples were tested for the Delta and Omicron variants. Patients who underwent COVID-19 without hospitalization were enrolled in the study. All participants were vaccinated with two doses of Pfizer vaccine. Subjects who became infected after at least 6 months after the second dose of the vaccine were selected for the study. The COVID-19 infection was confirmed by a documented positive RT-PCR test using a nasopharyngeal swab.

**Figure 1:**
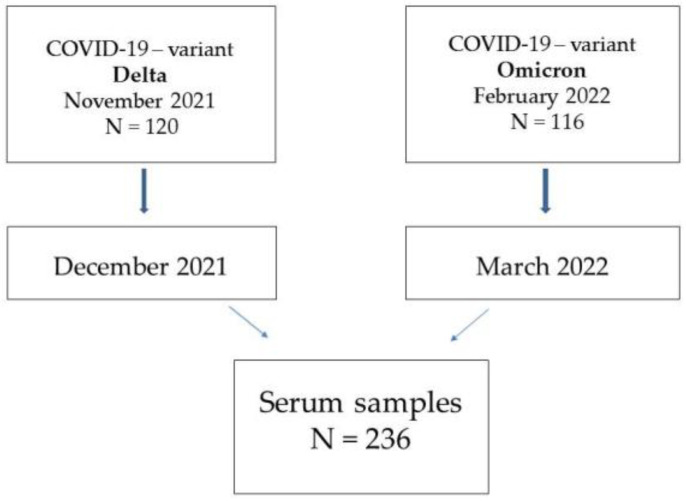
The scheme of study research.

Two age groups were selected among patients tested in our laboratory: 40–50 and 70–85. Infection with Delta and Omicron variants was also considered. The blood for testing the level of specific antibodies was collected from volunteers 2 months following the infection. The study population consisted of groups: infected with the Delta variant and infected with the Omicron variant. A total of 236 patients have been studied (Figure 1).

All participants answered a questionnaire regarding demographic, epidemiological, and clinical information and previous exposure to SARS-CoV-2.

### 2.2. Nasopharyngeal Swabs

#### 2.2.1. Detection of SARS CoV-2

The nasopharyngeal sample was previously extracted using an automated TANBead MaelstromTM 8 (TANBead Nucleic Acid Extraction Kit) and tested for SARS-CoV-2 at the SARS Laboratory of the Medical University in Lublin, Poland. The genesig^®^ Real-Time PCR Coronavirus COVID-19 (CE IVD) was used to detect SARS-CoV-2 viral RNA (Primerdesign Ltd., School Lane, Chandler’s Ford, Camberley, UK). The reaction system and amplification conditions were performed according to the manufacturer’s specifications. The result was considered positive when the cycle threshold (Ct) value of the viral gene was 38 or less, and negative when it was greater than 38. Coronavirus (COVID-19) CE IVD genesig^®^ kit detects 0.58 copies/μL of SARS-CoV-2 viral RNA with confidence ≥ 95%.

#### 2.2.2. Detection of SARS CoV-2 Variants

All positive RT-PCR assay samples were tested for variants of SARS-CoV-2. Delta and Omicron variants were detected using commercially available GSD NovaType IV SARS-CoV-2 RT-PCR kit (NovaTec Immundiagnostica GmbH, Dietzenbach, Germany, cat no PCOV6191T).

### 2.3. Serum Samples Collection

Venous blood samples (3–5 mL) from all subjects were taken by qualified and experienced laboratory staff. Tubes containing blood collected without anticoagulant were centrifuged at 1500× *g* rpm/15 min at room temperature, and then the serum was stored at 4 °C and used within five days.

#### Detection of SARS-CoV-2 Antibody

The serum samples from all individuals were tested using the Microblot-Array COVID-19 IgG assay (TestLine Clinical Diagnostics, Brno, Czech Republic) according to the manufacturer’s instructions. The Microblot-Array COVID-19 IgG assay detects the presence and titer of specific anti-SARS-CoV-2 antibodies: NCP, RBD, S2. The results are given in units of U/mL. The interpretation takes into account the presence or absence of a reaction against at least 1 antigen—NCP, RBD, or S2. The results were interpreted as follows: <185 U/mL = negative, 185–210 U/mL = borderline, >210 U/mL = positive.

### 2.4. Statistical Analysis

Results were analyzed using the Graph Pad Prism software version 9.4.1. The Mann–Whitney U test was used to analyze the antibody level depending on sex, age, and virus variant. Differences were considered statistically significant for *p* < 0.05.

### 2.5. Ethics

The research was approved by the Medical University of Lublin Ethics Committee and is in accordance with GCP regulations (no. KE-0254/121/2021, 27 May 2021). Written informed consent was obtained from each participant.

## 3. Results

### Characteristics of the Studied Population

Details of the characteristics of the studied group of 236 participants are presented in Table 1.

Out of 236 patients who underwent COVID-19 without hospitalization, 50.8% of patients (120 participants) were infected with the Delta variant and 49.2% (116 participants) with the Omicron variant (Table 1). In the whole group of patients, 50% (118 participants) were women and 50% were men. The subjects were divided into two age groups 40–50 (50%) and 70–85 (50%) (Table 1).

NCP, RBD, and S2 antibody titers were significantly lower in patients infected with Omicron variant: NCP—640.9 U/mL, RBD—886.0 U/mL, S2—746.8 U/mL compared to patients infected with Delta variant: NCP—438.1 U/mL, RBD—345.8 U/mL, S2—345.1 U/mL (Table 2).

**Figure 2:**
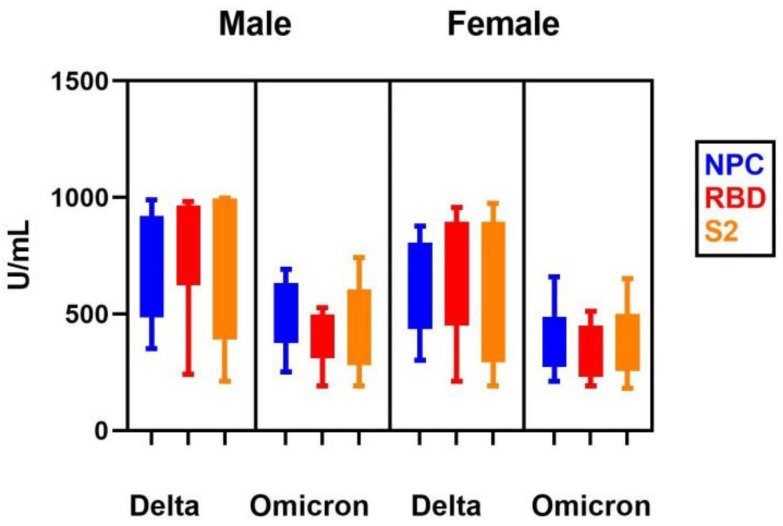
The serum level of NCP, RBD, and S2 antibody in patients after SARS CoV-2 Delta variant and Omicron variant infection by sex.

The level of NCP, RBD, and S2 antibodies in women and men after infection with the Delta variant did not differ. Similar results were obtained with the Omicron variant. However, both in men and women, analyzed antibodies after infection with the Omicron variant are statistically lower than after infection with the Delta variant (Figure 2).

**Figure 3:**
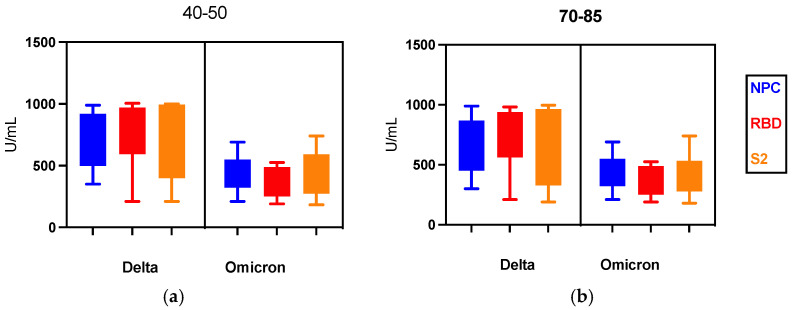
The serum level of NCP, RBD, and S2 antibody in patients after SARS CoV-2 Delta variant and Omicron variant infection by age: (**a**) 40–50, (**b**) 70–85.

There were no statistically significant differences in the levels of NCP, RBD, and S2 antibodies in the younger (40–50) and older (70–85) age groups. Antibody levels were similar in both age groups (Figure 3). In both age groups, the level of tested antibodies was higher in people infected with the Delta variant than in those infected with the Omicron variant.

Among men infected with the Delta variant, statistically, significantly higher levels of all tested antibodies were found in both tested age groups compared to the Omicron variant (Table 3). The level of NCP antibodies in the group of women did not differ depending on the age group. Among women in both studied age groups, the difference in the level of NCP antibodies was not statistically significant depending on the variant of SARS-CoV-2, Delta, or Omicron. However, the level of RBD and S2 antibodies in both age groups was higher in women infected with the Delta variant. This difference was statistically significant. Both in women and men in the younger age group (40–50), a slightly higher level of the tested NCP, RBD, and S2 antibodies was observed in comparison to the older age group (70–85) (Table 3).

## 4. Discussion

RNA viruses have a higher mutation rate than DNA viruses [14]. New variants still emerge in waves in different parts of the world causing similar or slightly different symptoms in patients and placing a smaller or greater burden on public healthcare systems [15,16].

SARS-CoV-2 mutations can affect not only the protein structure and replication cycle of virus, its infectivity, cytotoxicity, and immunogenicity, but also lead to false-negative diagnostic tests, reduced vaccine effectiveness, and the development of drug resistance. Furthermore, different variants have a distinct influence on the course of disease, causing mild or more severe symptoms [17].

Vaccinated people can be reinfected and it is called the breakthrough infection. According to the Food and Drug Administration, a COVID-19 vaccine breakthrough infection is defined as the detection of SARS-CoV-2 RNA or antigen in nasopharyngeal specimens collected from patients at least two weeks after full vaccination [18].

Several factors play a role in vaccines’ effectiveness; the difference in the heterogeneity of the immunized population, virus mutation, and the dominancy of a new variant of concern (VOC) which can decrease vaccine effectiveness [19]. Moreover, breakthrough infections demonstrate reduced vaccine effectiveness against B.1.617.2 [20,21,22,23].

Khan et al. [24] demonstrated that hybrid immunity after vaccination and Omicron BA.1 infection should be protective against Delta and other variants, whereas a single infection with only Omicron BA.1 gives limited cross-protection. Other authors showed that neutralizing antibody titers were lower for Omicron compared with the Delta variant [25]. Therefore, the effectiveness of the currently available SARS-CoV-2 vaccines against infection with the Omicron variant is lower compared to other SARS-CoV-2 variants [26,27].

Staerke et al. [28] described an association between higher levels of anti-spike antibodies and reduced risk of breakthrough infections for the Delta variant but not for the Omicron variant. Our patient group also acquired hybrid immunity. They were all vaccinated. Some of them were infected with the Delta variant and some with the Omicron variant. Our study showed how breakthrough infection affects antibody levels.

Various serological tests are available to detect SARS-CoV-2 antibodies. The virus neutralizing test (VNT) can determine the protective activity of antibodies. However, as demonstrated by Montesinos et al. [29,30], there is a significant degree of agreement in the IgG titers measured between Microblot-Array and the VNT. These authors compared the five different serology tests available to identify Nabs SARS-CoV-2 and concluded that Microblot-Array COVID-19 IgG assays could help assess possible waning loss of vaccine protection in the long term. The Microblot-Array COVID-19 IgG assay was used in our research.

We observed a statistically and significantly lower level of antibodies in patients infected with the Omicron variant compared to the individuals infected with the Delta variant. Therefore, these patients may be at risk of reinfection with a new Omicron variant. Recently, three lineages of Omicron variant, BA.1 (B.1.1.529.1), BA.2 (B.1.1.529.2), and BA.3 (B.1.1.529.3) have been described [31]. Omicron BA.1 has a total of 60 mutations in a non-structure protein (32 mutations inS protein, 15 mutations in RBD [32]. BA.2 shares 32 mutations with BA.1 but has 28 distinct ones. In RBD, BA.2 has four unique mutations and 12 shared with BA.1 [33,34].

New variants are also emerging in a poorly vaccinated population, so vaccination is important in preventing infection [10,11,12,35,36,37].

These new variants have the ability to spread rapidly [38]. Therefore, a decrease in humoral immunity may increase the risk of infection. For this reason, both PCR and rapid antigenic tests detect all Omicron subvariants and further tests are necessary to distinguish subvariants from each other and other SARS-CoV-2 variants. Due to the decrease in the titers of antibodies after vaccination, the possibility of reinfection, and too low levels of antibodies after infection with the Omicron variant, vaccination with a booster dose is recommended.

The limitation of our research is certainly too small group of patients. Unfortunately, not all patients pre-qualified for this analysis agreed to participate in the study. Nevertheless, further epidemiological and clinical studies are necessary to develop new prevention strategies.

## 5. Conclusions

The level of antibodies in patients after SARS CoV-2 Omicron variant infection was lower than after Delta infection. Due to the possibility of reinfection, booster vaccinations are necessary.

## Figures and Tables

**Table 1:** Characteristics of studied individuals.

Characteristics		N	%
Sex	Female	118	50.0
	Male	118	50.0
Age	40–50	118	50.0
	70–85	118	50.0
Past COVID-19	Delta	120	50.8
	Omicron	116	49.2

**Table 2:** The level of NCP, RBD, and S2 (U/mL) in studied groups by SARS-CoV-2 variant.

Patients’ Group	NCP	RBD	S2
COVID-19 Delta	640.9 (300.7–989.3)	886.0 (210.1–1000.1)	746.8 (190.7–1000.0)
COVID-19 Omicron	438.1 (210.0–690.7)	345.8 (190.1–512.1)	345.1 (180.1–740.5)
*p*	10^−6^ *	10^−6^ *	10^−6^ *

Mann–Whitney U Test; Median (min-max); * statistically significant.

**Table 3:** The level of NCP, RBD, and S2 (U/mL) by age, sex, and SARS CoV-2 variant.

Patients’ Group	NCP		RBD		S2	
	Delta	Omicron	Delta	Omicron	Delta	Omicron
Male						
40–50	717.3 (350.7–989.3)	485.4 (250.8–690.7)	948.7 (240.9–1005.5)	456.2 (190.5–525.7)	852.8 (210.7–1000.0)	361.4 (190.6–740.5)
*p*	0.001 *		0.001 *		0.001 *	
70–85	667.3 (350.7–989.3)	485.4 (250.8–690.7)	842.7 (240.9–982.3)	456.2 (190.3–526.1)	723.5 (210.7–997.1)	361.5 (190.5–740.3)
*p*	0.001 *		0.001 *		0.001 *	
Female						
40–50	497.5 (350.7–989.3)	390.7 (210.0–659.0)	842.1 (210.1–1005.0)	345.8 (190.1–512.1)	723.4 (210.7–1000.0)	314.8 (180.5–650.6)
*p*	>0.05		0.001 *		0.001 *	
70–85	439.7 (300.9–878.7)	390.6 (210.0–658.1)	790.0 (210.4–956.4)	345.5 (190.0–510.1)	606.7 (190.7–975.8)	340.0 (180.5–650.3)
*p*	>0.05		0.001 *		0.001 *	

Mann–Whitney U Test; Median (min-max); * Statistically significant.

## Data Availability

The data presented in this study are available in the article.
